# Periodontal health in a cohort of subjects with type 1 diabetes mellitus

**DOI:** 10.1002/cre2.178

**Published:** 2019-03-09

**Authors:** Margaux Roy, Giacomo Gastaldi, Delphine S. Courvoisier, Andrea Mombelli, Catherine Giannopoulou

**Affiliations:** ^1^ Division of Periodontology University Clinics of Dental Medicine, University of Geneva Geneva Switzerland; ^2^ Diabetology Unit University Hospitals of Geneva Geneva Switzerland; ^3^ Division of Rheumatology University Hospitals of Geneva Geneva Switzerland

**Keywords:** age, oral health habits, periodontal disease, type 1 diabetes

## Abstract

To evaluate periodontal health and oral health behaviors in a cohort of subjects with type 1 diabetes (T1D), 50 persons with T1D (30 males and 20 females; mean age: 35.2 years) were recruited from the Diabetology Unit of the Geneva University Hospitals; 50 nondiabetic persons matched for gender, age, and smoking status comprised the control group. We assessed periodontal health using the gingival index (GI), plaque index, probing depth (PD), bleeding on probing (BOP), and clinical attachment level (CAL) and recorded self‐reported attitudes and behaviors regarding dental care. The two groups were compared using conditional logistic regression. With respect to the mean PD, CAL, and the mean number of sites with PD >4 mm that bled upon probing, there were no significant differences between the groups. However, subjects with diabetes had significantly more plaque and gingival inflammation and presented more sites with BOP compared with control subjects. Further analysis of the subjects in younger (<40 years) and older (>40 years) cohorts revealed a marked difference in GI between younger healthy and controls, which was also present in older patients and controls but much reduced in magnitude and significance. This marked difference in the gingival health of young versus old diabetic patients to matched controls may provide diagnostic advantages and screening and prevention opportunities to exploit. In spite of similar self‐reported oral hygiene habits and frequency of dental visits, patients with T1D presented more plaque and more inflammation than healthy controls, particularly in the younger subjects. Gingivitis in young T1D patients may be an early indicator for more complicated diabetes and periodontitis in the future. Thus, patients with T1D mellitus should be screened for signs of periodontal disease early and should be motivated and instructed in good oral hygiene practices.

## 1 INTRODUCTION

Gingivitis and periodontitis are chronic inflammatory diseases that affect the supporting tissues of the teeth. Although induced by the presence of bacterial biofilms on the teeth (Sanz et al., 2017), other factors, such as tobacco smoking, drugs, immunodeficiency, and various systemic diseases, are known to influence their pathogenesis. Diabetes mellitus (DM) is an established factor with a significant impact on prevalence, severity, and progression of periodontitis (Atieh, Faggion, & Seymour, 2014; Mealey & Oates, 2006; Taylor & Borgnakke, 2008). Two types of diabetes are recognized: Type 1 diabetes (T1D) results from the autoimmune destruction of the insulin‐producing pancreatic islet cells leading to loss of insulin production; type 2 diabetes (T2D) results from resistance to insulin and relative lack of insulin. In both cases, chronic hyperglycemia is established, which in turn enhances oxidative stress in periodontal tissues, dysregulates cytokine production, and promotes inflammation via the systemic accumulation of advanced glycation end products (AGEs). AGEs induce an increased expression of cell surface receptors for AGE leading to a more pronounced cell stress (Abbass, Korany, Salama, Dmytryk, & Safiejko‐Mroczka, 2012; Lalla, Lamster, Drury, Fu, & Schmidt, 2000). The presence of pathogenic bacteria further enhances cell stress (Chapple & Genco, 2013).

A bidirectional relationship between T2D and periodontitis has been documented in several studies: Not only does diabetes adversely affect periodontal conditions but periodontitis, especially the severe form, also adversely affects the glycemic control in diabetic patients (Chavarry, Vettore, Sansone, & Sheiham, 2009; Lalla & Papapanou, 2011). Significantly less studies have focused on the association between periodontal health and T1D. It comprises 5–10% of the diabetic subjects with a rise in prevalence of more than 1.4% each year in the western world (Mayer‐Davis et al., 2018). The diagnosis is usually established at a young age, although recent epidemiological studies have shown that T1D can onset also in adults and even at advanced age (Mayer‐Davis et al., 2018). A systematic review reported poorer periodontal conditions in children with T1D, as compared with systemically healthy children, notably with regards to greater plaque accumulation and higher levels of inflammation (Ismail, McGrath, & Yiu, 2015). For caries experience, the evidence was inconclusive. A population‐based prospective cohort study in East Germany examined the influence of T1D and T2D on periodontal disease progression over 5 years (Demmer et al., 2012). T1D subjects were from 20 to 81 years of age. The authors reported a direct influence of uncontrolled diabetic status on progression of attachment loss for both disease types.

The prognostic value of various clinical parameters to indicate disease progression has been evaluated in clinical studies (Gonzalez et al., 2015; Lang, Schatzle, & Loe, 2009; Schatzle et al., 2003). Among these, bleeding on probing (BOP) has been associated to higher risk for future attachment loss: Sites that repeatedly bled on probing had a significantly higher risk for attachment loss compared with sites with no BOP (Schatzle et al., 2003). These studies showed that periodontitis only occurs in areas of long‐standing gingivitis, suggesting that gingivitis is an obligatory precursor of periodontitis.

The accumulation of bacterial biofilms in T1D subjects may lead to more severe gingivitis, which in turn may increase the risk to develop periodontitis. In the present case–control study, we assessed the periodontal conditions and oral health behaviors of a cohort of subjects with T1D and compared them with those of a group of age‐ and gender‐matched nondiabetic individuals. In the T1D group, we further analyzed the impact of diabetes‐related factors, such as the duration of the diabetes and the number of complications on the periodontal conditions.

## 2 MATERIAL AND METHODS

### 2.1 Study design

This was a single‐center, cross‐sectional study. The Ethical Committee of the University Hospitals of Geneva, Geneva, Switzerland, approved the protocol. All participants gave written informed consent. From July 2016 to July 2018, 72 individuals with T1D were contacted from the patient cohort of the Diabetology Unit of the Geneva University Hospitals. Among these, 50 subjects, aged between 18 and 85 years, agreed to have dental examination for the purpose of the present study. For the remaining 22 subjects, the main reason for refusal was the long distance to the dental clinic. For inclusion, subjects had to be diagnosed with T1D for more than 1 year and have at least 10 natural remaining teeth. Subjects with a history of systemic disease such as cancer, HIV, bone metabolic disease, disorders that compromise wound healing, history of radiation, or immunosuppressive/modulating therapy were excluded, as well as those who had taken antibiotics in the previous 3 months or NSAIDs in the previous 2 months. Fifty periodontally healthy controls matched for age, sex, and smoking status were recruited among patients attending the School of Dental Medicine of the University of Geneva. The matching for smoking was achieved by the self‐reported smoking habits (number of cigarettes per day and number of years) from the participants.

### 2.2 Medical visit

During a routine medical visit at the Diabetology Clinic, data concerning the date of diagnosis, presence of specific antibodies related to T1D, mean glycated hemoglobin in the past 3 years, body mass index, number of diabetes complications (retinopathy, nephropathy, neuropathy, and macrovascular complications), kind of glucose monitoring (self‐monitoring blood glucose or continuous glucose monitoring system or flash glucose monitoring), and insulin route of administration (insulin pump or multiple daily injections) were collected.

In addition, in a subsample of 28 patients, electrochemical skin conductance (ESC) was measured by Sudoscan, a noninvasive tool for detecting diabetic small fiber neuropathy. ESC values are expressed in microSiemens (μS) and registered with plate electrodes placed on the subject's hands and feet, to measure sweat gland function (Casellini, Parson, Richardson, Nevoret, & Vinik, 2013). An ESC value of >60 μS corresponds to no dysfunction, 60–40 μs corresponds to moderate dysfunction, and <40 μS to severe dysfunction.

### 2.3 Periodontal examination

Before the periodontal examination, information regarding dental history was obtained by questionnaire and included oral hygiene habits, frequency of dental appointments, smoking habits, and previous dental treatments. A single experienced examiner (M. R.) performed a full‐mouth clinical examination. The examiner was not blind concerning the diabetes status. The following clinical parameters were recorded at six sites per tooth of each subject: plaque index (Silness & Löe, 1964), gingival index (GI; Löe & Silness, 1963), probing pocket depth (PD) using a manual probe, BOP, gingival recession (REC), furcation involvement, and tooth mobility. The clinical attachment level (CAL) of each site was calculated as PD + REC.

The severity of periodontitis was graded according to the CDC/AAP classification (Albandar, 2007) as follows: mild periodontitis, presence of one or more teeth with interproximal sites showing ≥4‐mm CAL and ≥4‐mm PD; moderate periodontitis, presence of two or more nonadjacent teeth with interproximal sites showing ≥5‐mm CAL and ≥4‐mm PD; severe periodontitis, presence of two or more nonadjacent teeth with interproximal sites showing ≥6‐mm CAL and ≥4‐mm PD. For the participants with no signs of attachment loss, the diagnosis of gingivitis was attributed when the BOP score was >10%, and healthy periodontal status was attributed when the BOP score was <10% (Chapple et al., 2018).

For subjects of the control group, absence of diabetes was confirmed by an HbA1c test. The system used (A1C Now+, pts Diagnostics) provided the results in a few minutes using blood, sampled with a fingerstick.

### 2.4 Statistical analysis

Diabetic and nondiabetic participants were described using frequencies and percentages for categorical variables and mean and standard deviations for continuous variables. The two groups were compared using conditional logistic regression. We used the same tests for comparisons when each group (diabetic and nondiabetic) was further divided in younger (<40 years old) and older (≥40 years old) subjects. Prediction of periodontal status was done using mixed effects logistic regression to account for the matched structure of the data. All analyses were conducted using R v3.5.1, with a significance threshold set at *P* < 0.05.

## 3 RESULTS

Fifty subjects with T1DM and 50 nondiabetic subjects matched for gender, age, and smoking status participated in the study. The mean age was 35 years; and approximately 40% of the subjects of both groups were females. As shown in Table 1, the 3‐year mean HbA1c in the diabetic group was 8.3% and for the nondiabetic group 5.2%, the difference being statistically significant (*P* < 0.001). The overall characteristics of the diabetic population are shown in Table 2. The mean time since diagnosis of diabetes was 13.3 years (±11.9), and T1D subjects had a mean body mass index of 24.5 (±3.8). The Sudoscan measurements showed that only one among the 28 subjects presented severe dysfunction in the right hand.

**Table 1:** Characteristics of the study population

	Control	Diabetic	*P*
Gender			1.000
Male	30 (60)	30 (60)	
Female	20 (40)	20 (40)	
Age (years), mean ± *SD*	35.9 (15.0)	35.2 (15.0)	0.020
HbA1c (%),mean ± *SD*	5.2 (0.4)	8.3 (1.8)	<0.001
Smoking			0.958
Never	27 (54.0)	26 (52.0)	
Light smoker (<10/day)	7 (14.0)	7 (14.0)	
Heavy smoker (≥10/day)	5 (10.0)	5 (10.0)	
e‐cigarette	2 (4.0)	1 (2.0)	
Former	9 (18.0)	11 (22.0)	
UPA, mean ± *SD*	5.8 (8.8)	6.3 (9.0)	

**Table 2:** Background characteristics for diabetic subjects

	Value
Duration of diabetes (years), mean ± *SD*	13.3 (11.9)
BMI, mean ± *SD*	24.5 (3.8)
Administration of insulin (%)	
MDI (multiple daily injections)	28 (58.0)
Pump	21 (42.0)
Glucose monitoring	
No monitoring	9 (18.0)
FGM (flash glucose monitoring)	11 (22.0)
CGM (continuous glucose monitoring	30 (60.0)
Complications	
Number of complication, mean ± *SD*	0.6 (1.0)
Retinopathy	11 (22.0)
Nephropathy	7 (14.0)
Neuropathy	7 (14.0)
Cardiovascular disease	1 (2.0)
SNC	0
IAMI	0
Sudoscan	
Right hand, <40 μSv	1 (3.6)
Right hand, 40‐60 μSv	4 (14.3)
Left hand, 40‐60 μSv	3 (10.7)
Right foot, 40‐60 μSv	1 (3.6)
Left foot, 40‐60 μSv	1 (3.6)

The oral health behavior of the study populations is shown in Table 3. The frequency of tooth brushing varied between once a day (9%), twice (34%), and more than twice a day (7%) for both groups; 60% of the diabetics and 40% of the nondiabetics never performed approximal tooth cleaning; the respective values for once a day was 14% and 26%, whereas for once a week, it was 26% and 34%. Subjects of both groups reported having regular dental appointments at least once per year. However, 24% of the subjects in both groups reported having an appointment only in case of emergency. In the same table, the previous dental treatments are shown. An important number of diabetics (30%) and nondiabetics (46%) had received orthodontic treatment in the past.

**Table 3:** Self‐reported oral health habits and dental history

	Control	Diabetic	*P*
Tooth brushing, *N* (%)			1.000
Once a day	10 (20.0)	8 (16.0)	
Twice a day	32 (64.0)	36 (72)	
>2 times a day	8 (16.0)	6 (12)	
Approximal tooth cleaning, *N* (%)			0.062
Never	20 (40.0)	30 (60.0)	
Once a week	17 (34.0)	13 (26.0)	
Once a day	13 (26.0)	7 (14.0)	
Frequency of dental recalls, *N* (%)			0.426
Never	12 (24.0)	12 (24.0)	
Once a year	32 (64.0)	27 (54.0)	
Twice a year	4 (8.0)	8 (16.0)	
>2 times a year	2 (4.0)	3 (6.0)	
Dental treatments			
Periodontal	6 (12.0)	5 (10.0)	0.706
Dental implants	5 (10.0)	3 (6.0)	0.484
Orthodontic	23 (46.0)	15 (30.0)	0.082
Contention	19 (38.0)	9 (18.0)	**0.027**

Table 4 shows the clinical characteristics of the study populations. The number of teeth present, the mean PD, REC, AL, and the mean number of sites with a plaque index score of ≥1 and the mean number of sites with PD > 4 mm that bled upon probing did not differ between the groups. All the other clinical parameters, including the mean presence of plaque, GI, BOP, and the mean number of sites with GI score ≥ 1, were significantly higher in the diabetic as compared with nondiabetic group.

**Table 4:** Dental examination results

	Control	Diabetic	*P*
Number of teeth, % ± *SD*	26.2 (2.8)	26.8 (2.6)	0.248
PI, % ± *SD*	0.4 (0.2)	0.5 (0.4)	0.014
GI, % ± *SD*	0.4 (0.4)	1.1 (0.7)	0.000
BOP, % ± *SD*	29.4 (16.4)	40.5 (22.2)	0.009
PD, % ± *SD*	2.5 (0.3)	2.5 (0.4)	0.381
Recession, % ± *SD*	0.2 (0.2)	0.3 (0.3)	0.083
CAL, % ± *SD*	2.6 (0.4)	2.8 (0.6)	0.070
Number of sites PI > 1, % ± *SD*	13.8 (14.5)	23.9 (27.2)	0.047
Number of sites GI > 1, % ± *SD*	18.8 (23.1)	59.2 (57.6)	0.001
Number of sites PD > 4 + BOP	1.5 (3.7)	2.3 (5.0)	0.336

*Note*. BOP: bleeding on probing; GI: gingival index; PD: probing depth; PI: plaque index.

Concerning the diagnosis of periodontal disease, no significant differences were observed between the groups. As shown in Table 5, gingivitis was present in 68% of the diabetics and 60% of the nondiabetics. Fifteen diabetic subjects (30%) and 14 (35%) nondiabetics had a diagnosis of periodontitis according to the CDC/AAP classification. We further compared periodontal parameters between controls and diabetics in younger (<40 years old) and older (>40 years old) subjects. As shown in Table 6, diabetics <40 years old had significantly more plaque (*P* = 0.004) and more inflammation (GI; *P* < 0.001) compared with their matched controls. In the older group (>40 years old), only gingival inflammation was significantly higher in diabetics compared with controls (*P* = 0.003). The associations among several measured parameters that affect the periodontal condition are shown in Table 7A. The only variables identified as determinants of the periodontal conditions in the whole group (both diabetic and control) were age (*P* < 0.001), BOP (*P* = 0.009), and smoking (*P* = 0.01). However, when considering these three factors together, only age and BOP remained significantly associated with periodontitis (Table 7A). Finally, when examining the associations of the parameters with periodontitis only among diabetic patients, age, HbA1c, BOP, and smoking were significantly associated with periodontitis (Table 7B).

**Table 5:** Periodontal status of the study population

	Control	Diabetic	*P*
			0.258
Healthy periodontium	6 (12.0)	1 (2.0)	
Gingivitis	30 (60.0)	34 (68.0)	
Periodontitis			
Mild periodontitis	7 (14.0)	6 (12.0)	
Moderate periodontitis	2 (5.0)	5 (10.0)	
Severe periodontitis	5 (10.0)	4 (8.0)	

**Table 6:** Periodontal parameters in controls and diabetics <40 years and > 40 years old

	Control (*n* = 28) <40 years old	Diabetic (*n* = 28)	*P*	Control (*n* = 22) ≥40 years old	Diabetic (*n* = 22)	*P*
PI, mean ± *SD*	0.3 (0.2)	0.6 (0.4)	0.004	0.4 (0.3)	0.5 (0.4)	0.260
GI, mean ± *SD*	0.3 (0.3)	1.1 (0.7)	0.000	0.5 (0.4)	1.0 (0.6)	0.003
BOP, % ± *SD*	32 (43.4)	41 (23.6)	0.342	37 (19.3)	40 (20.7)	0.607
PD, mean ± *SD*	2.2 (0.5)	2.4 (0.2)	0.043	2.7 (0.3)	2.6 (0.5)	0.796

*Note*. BOP: bleeding on probing; GI: gingival index; PD: probing depth; PI: plaque index.

**Table 7A:** Mixed effects logistic regression for odds of periodontitis

	Univariable OR [95% CI]	*P*	Adjusted OR [95% CI]	*P*
Diabetes status	1.13 [0.42, 3.10]	0.83		
Age	1.10 [1.05, 1.17]	<0.001	1.09 [1.04, 1.18]	0.002
HbA1c	0.88 [0.65, 1.15]	0.36		
BOP	1.04 [1.02, 1.09]	0.009	1.04 [1.01, 1.08]	0.02
Brushing at least twice a day	0.37 [0.09, 1.40]	0.15		
Current smoking	4.70 [1.57, 19.81]	0.01	2.41 [0.75, 9.66]	0.14
Dental recall	1.08 [0.52, 2.27]	0.84		
Number of complications	1.06 [0.51, 2.06]	0.86		

*Note*. BOP: bleeding on probing.

**Table 7B:** Mixed effects logistic regression for odds of periodontitis among diabetic patients

	Univariable OR [95% CI]	*P*
Age	1.09 [1.04, 1.16]	0.003
HbA1c	0.53 [0.27, 0.89]	0.04
Diabetes duration	0.95 [0.88, 1.01]	0.15
BOP	1.03 [1.00, 1.06]	0.048
Brushing at least twice a day	0.35 [0.07, 1.73]	0.19
Current smoking	5.06 [1.42, 19.7]	0.01
Dental recall	0.78 [0.34, 1.67]	0.54
Number of complications	1.12 [0.60, 1.98]	0.70

*Note*. BOP: bleeding on probing.

## 4 DISCUSSION

The aim of the present cross‐sectional, case–control study was to evaluate the periodontal clinical conditions and oral health behavior in a cohort of subjects with T1D and in a control group matched for age, sex, and smoking status. Results showed that T1DM subjects presented significantly more plaque and more inflammation as compared with the control group in spite of similar self‐reported oral hygiene habits and frequency of dental visits. However, the prevalence of periodontitis did not differ between the two groups. Multivariable logistic regression showed that periodontitis was related mainly to age and BOP index. Our hypothesis was based on the findings of the longitudinal studies of Lang et al. (2009) and Schatzle et al. (2003), who reported that gingivitis precedes the established periodontal lesion and thus can be considered as a risk factor in periodontal disease. In their studies, teeth scored GI = 0 had a mean cumulative attachment loss (LA) of <2 mm over 60 years life span, teeth with slight inflammation (GI = 1) had a mean LA of >2 mm, and those who consistently bled on probing (GI = 2), the mean LA was >3 mm. In the present study, the higher inflammation, in terms of GI and BOP scores found in the diabetic group, suggests that these subjects will be more susceptible in developing periodontitis in the future. Furthermore, the high oral hygiene level of the individuals further confirms that the higher inflammation is not related to simply poor oral hygiene habits but is an innate susceptibility feature of the patient. An interesting finding in the present study was the marked difference in GI for the younger cohort, supporting a preventive approach with good diagnostic differentiation possibilities and strong treatment opportunities in the younger age group. The reduction in differences between older diabetic and matched nondiabetic subjects may reflect a plateauing of the inflammatory burden on the gingiva with aging.

The association between T1DM and oral health conditions and the assumption that T1DM is a risk factor for periodontitis has been the subject of several investigations.

Two previously published systematic reviews and meta‐analysis concluded that the evidence of a link between T1DM and periodontitis is not sufficient (Chavarry et al., 2009; Khader, Dauod, El‐Qaderi, Alkafajei, & Batayha, 2006). However, the studies included in these reviews had several important drawbacks, such as small sample size, control group not matched for age, gender, or other parameters, periodontal measures recorded to half of the mouth, lack of the examiners' calibration, and lack of taking in consideration potential confounding factors.

More recently, two cross‐sectional studies, including subjects from five hospitals in Glasgow, reported that the prevalence of severe periodontitis, in terms of clinical attachment loss and radiographic bone loss, was significantly higher in both well‐controlled and poorly controlled subjects with T1DM as compared with nondiabetic subjects (Hodge et al., 2012; Plessas, Robertson, & Hodge, 2018). In a cohort of subjects with T1DM, the bacterial profile based on 12 species was examined and was compared with that from a control group matched for age, gender, and level of periodontitis. No significant difference was observed between the two groups, suggesting that it is the host response to the bacterial challenge that drives the enhanced susceptibility to periodontal disease in diabetes (Lalla et al., 2006).

The majority of the studies emphasized that the duration of diabetes, poor metabolic control, and other existing complications of diabetes are important factors to take into consideration in the evaluation of diabetes as a risk factor for periodontal disease. In our study, the prevalence of periodontitis—mild, moderate, or severe—did not differ between the diabetic and nondiabetic population. It should be emphasized, however, that the majority of the participants were nonsmokers or former smokers (53% and 20%, respectively) and did not have any severe complications related to their diabetes status. Indeed, only one subject out of 50 diabetics had a major complication affecting the microvasculature (cardiovascular disease), seven suffered from neuropathy, seven others from nephropathy, and 11 subjects from retinopathy. In addition, the majority of subjects reported having annual dental appointment. More specifically, subjects in the control group were recruited among patients of the dental school who regularly see the students and receive repeatedly oral hygiene instructions.

In Switzerland, a population‐based cross‐sectional survey was conducted in the canton de Vaud in order to assess the quality of care provided to patients with diabetes. Based on self‐administered paper questionnaires, among the 406 participants, 18.2% had T1DM, 68.5% had T2DM, and for 20% of the subjects, the diabetes type remained undetermined. Although routine clinical and laboratory tests were performed annually in most of the subjects, several risk screenings related to diabetes were less often reported. For example, feet examination, microalbuminuria, and physical activity and dietary recommendations were reported only by a minority of the subjects (Peytremann‐Bridevaux, Bordet, & Burnand, 2013). Subjects had received no recommendation for dental examination.

In conclusion, the results of this study add to the body of literature supporting the observation that there is a high prevalence of plaque and gingival inflammation in subjects with T1DM. Whether these subjects are more susceptible to develop severe forms of periodontal disease in the future remains to be elucidated longitudinally. Our further analysis of the subjects in younger (<40 years) and older (>40 years) T1DM cohorts revealed a marked difference in GI between younger healthy and controls, which was less pronounced in older patients. This marked difference in the gingival health of young versus old diabetic patients to matched controls may provide diagnostic advantages and screening and prevention opportunities to exploit. We suggest that periodontal health, particularly gingivitis in younger patients, may be an early indicator for both more complicated diabetes and periodontitis, and thus, oral health education and early diagnosis and treatment of periodontal disease should be recommended by both physicians and dentists to T1DM subjects.

## CONFLICT OF INTEREST

The authors declare no conflict of interest.

## References

[cre2178-bib-0001] Abbass, M. M. , Korany, N. S. , Salama, A. H. , Dmytryk, J. J. , & Safiejko‐Mroczka, B. (2012). The relationship between receptor for advanced glycation end products expression and the severity of periodontal disease in the gingiva of diabetic and non diabetic periodontitis patients. Archives of Oral Biology, 57, 1342–1354. 10.1016/j.archoralbio.2012.06.007 22795565

[cre2178-bib-0002] Albandar, J. M. (2007). Periodontal disease surveillance. Journal of Periodontology, 78, 1179–1181. 10.1902/jop.2007.070166 17608570

[cre2178-bib-0003] Atieh, M. A. , Faggion, C. M. Jr. , & Seymour, G. J. (2014). Cytokines in patients with type 2 diabetes and chronic periodontitis: A systematic review and meta‐analysis. Diabetes Research and Clinical Practice, 104, e38–e45. 10.1016/j.diabres.2014.02.002 24655616

[cre2178-bib-0004] Casellini, C. M. , Parson, H. K. , Richardson, M. S. , Nevoret, M. L. , & Vinik, A. I. (2013). Sudoscan, a noninvasive tool for detecting diabetic small fiber neuropathy and autonomic dysfunction. Diabetes Technology & Therapeutics, 15, 948–953. 10.1089/dia.2013.0129 23889506 PMC3817891

[cre2178-bib-0005] Chapple, I. L. , & Genco, R. (2013). Diabetes and periodontal diseases: Consensus report of the Joint EFP/AAP Workshop on Periodontitis and Systemic Diseases. Journal of Clinical Periodontology, 40(Suppl 14), S106–S112. 10.1111/jcpe.12077 23627322

[cre2178-bib-0006] Chapple, I. L. C. , Mealey, B. L. , Van Dyke, T. E. , Bartold, P. M. , Dommisch, H. , Eickholz, P. , … Griffin, T. J. (2018). Periodontal health and gingival diseases and conditions on an intact and a reduced periodontium: Consensus report of workgroup 1 of the 2017 World Workshop on the Classification of Periodontal and Peri‐Implant Diseases and Conditions. Journal of Clinical Periodontology, 45(Suppl 20), S68–s77. 10.1111/jcpe.12940 29926499

[cre2178-bib-0007] Chavarry, N. G. , Vettore, M. V. , Sansone, C. , & Sheiham, A. (2009). The relationship between diabetes mellitus and destructive periodontal disease: A meta‐analysis. Oral Health & Preventive Dentistry, 7, 107–127. 19583037

[cre2178-bib-0008] Demmer, R. T. , Holtfreter, B. , Desvarieux, M. , Jacobs, D. R. , Kerner, W. , Nauck, M. , … Kocher, T. (2012). The influence of type 1 and type 2 diabetes on periodontal disease progression: Prospective results from the Study of Health in Pomerania (SHIP). Diabetes Care, 35, 2036–2042. 10.2337/dc11-2453 22855731 PMC3447825

[cre2178-bib-0009] Gonzalez, S. , Cohen, C. L. , Galvan, M. , Alonaizan, F. A. , Rich, S. K. , & Slots, J. (2015). Gingival bleeding on probing: Relationship to change in periodontal pocket depth and effect of sodium hypochlorite oral rinse. Journal of Periodontal Research, 50, 397–402. 10.1111/jre.12219 25040766

[cre2178-bib-0010] Hodge, P. J. , Robertson, D. , Paterson, K. , Smith, G. L. , Creanor, S. , & Sherriff, A. (2012). Periodontitis in non‐smoking type 1 diabetic adults: A cross‐sectional study. Journal of Clinical Periodontology, 39, 20–29. 10.1111/j.1600-051X.2011.01791.x 22092931

[cre2178-bib-0011] Ismail, A. F. , McGrath, C. P. , & Yiu, C. K. (2015). Oral health of children with type 1 diabetes mellitus: A systematic review. Diabetes Research and Clinical Practice, 108, 369–381. 10.1016/j.diabres.2015.03.003 25817182

[cre2178-bib-0012] Khader, Y. S. , Dauod, A. S. , El‐Qaderi, S. S. , Alkafajei, A. , & Batayha, W. Q. (2006). Periodontal status of diabetics compared with nondiabetics: A meta‐analysis. Journal of Diabetes and its Complications, 20, 59–68. 10.1016/j.jdiacomp.2005.05.006 16389170

[cre2178-bib-0013] Lalla, E. , Kaplan, S. , Chang, S. M. , Roth, G. A. , Celenti, R. , Hinckley, K. , … Papapanou, P. N. (2006). Periodontal infection profiles in type 1 diabetes. Journal of Clinical Periodontology, 33, 855–862. 10.1111/j.1600-051X.2006.00996.x 17092237

[cre2178-bib-0014] Lalla, E. , Lamster, I. B. , Drury, S. , Fu, C. , & Schmidt, A. M. (2000). Hyperglycemia, glycoxidation and receptor for advanced glycation endproducts: Potential mechanisms underlying diabetic complications, including diabetes‐associated periodontitis. Periodontology, 2000(23), 50–62. 10.1034/j.1600-0757.2000.2230104.x 11276765

[cre2178-bib-0015] Lalla, E. , & Papapanou, P. N. (2011). Diabetes mellitus and periodontitis: A tale of two common interrelated diseases. Nature Reviews Endocrinology, 7, 738–748. 10.1038/nrendo.2011.106 21709707

[cre2178-bib-0016] Lang, N. P. , Schatzle, M. A. , & Loe, H. (2009). Gingivitis as a risk factor in periodontal disease. Journal of Clinical Periodontology, 36(Suppl 10), 3–8. 10.1111/j.1600-051X.2009.01415.x 19432625

[cre2178-bib-0017] Löe, H. , & Silness, J. (1963). Periodontal disease in pregnancy. I. Prevalence and severity. Acta Odontologica Scandinavica, 21, 533–551. 10.3109/00016356309011240 14121956

[cre2178-bib-0018] Mayer‐Davis, E. J. , Kahkoska, A. R. , Jefferies, C. , Dabelea, D. , Balde, N. , Gong, C. X. , … Craig, M. E. (2018). ISPAD Clinical Practice Consensus Guidelines 2018: Definition, epidemiology, and classification of diabetes in children and adolescents. Pediatric Diabetes, 19(Suppl 27), 7–19. 10.1111/pedi.12773 PMC7521365 30226024

[cre2178-bib-0019] Mealey, B. L. , & Oates, T. W. (2006). Diabetes mellitus and periodontal diseases. Journal of Periodontology, 77, 1289–1303. 10.1902/jop.2006.050459 16881798

[cre2178-bib-0020] Peytremann‐Bridevaux, I. , Bordet, J. , & Burnand, B. (2013). Diabetes care in Switzerland: Good, but perfectible: A population‐based cross‐sectional survey. BMC Health Services Research, 13, 232 10.1186/1472-6963-13-232 23800376 PMC3722105

[cre2178-bib-0021] Plessas, A. , Robertson, D. P. , & Hodge, P. J. (2018). Radiographic bone loss in a Scottish non‐smoking type 1 diabetes mellitus population: A bitewing radiographic study. Journal of Periodontology, 89, 1043–1051. 10.1002/JPER.16-0788 29766516

[cre2178-bib-0022] Sanz, M. , Beighton, D. , Curtis, M. A. , Cury, J. A. , Dige, I. , Dommisch, H. , … Könönen, E. (2017). Role of microbial biofilms in the maintenance of oral health and in the development of dental caries and periodontal diseases. Consensus report of group 1 of the Joint EFP/ORCA workshop on the boundaries between caries and periodontal disease. Journal of Clinical Periodontology, 44(Suppl 18), S5–s11. 10.1111/jcpe.12682 28266109

[cre2178-bib-0023] Schatzle, M. , Loe, H. , Burgin, W. , Anerud, A. , Boysen, H. , & Lang, N. P. (2003). Clinical course of chronic periodontitis. I. Role of gingivitis. Journal of Clinical Periodontology, 30, 887–901. 10.1034/j.1600-051X.2003.00414.x 14710769

[cre2178-bib-0024] Silness, J. , & Löe, H. (1964). Periodontal disease in pregnancy. II. Correlation between oral hygiene and periodontal condition. Acta Odontologica Scandinavica, 22, 121–135. 10.3109/00016356408993968 14158464

[cre2178-bib-0025] Taylor, G. , & Borgnakke, W. (2008). Periodontal disease: Associations with diabetes, glycemic control and complications. Oral Diseases, 14, 191–203. 10.1111/j.1601-0825.2008.01442.x 18336370

